# The communication path and improvement strategy of symbolic culture of sneaker consumption culture using the big data analysis

**DOI:** 10.1371/journal.pone.0287757

**Published:** 2023-07-19

**Authors:** Zhongyuan Lv

**Affiliations:** School of Law, Hohai University, Nanjing, China; King Khalid University, SAUDI ARABIA

## Abstract

With the emergence of Artificial Intelligence technology and the advancement of science and technology, the current mainstream path of social development is continuously updating and improving various industries using technology. Therefore, in order to promote the development of sneaker consumer culture, this study explores the use of technological means to improve the dissemination effect of symbolic culture in sneaker consumer culture. Firstly, the development concept and mainstream direction of sneaker consumer culture in the era of big data are discussed, and the application principle of big data technology is introduced. Then, a sneaker culture dissemination model based on big data technology is designed. Finally, the model is optimized using a Convolutional Neural Network (CNN), and its effectiveness is evaluated. The results show that the Convolutional Neural Network-Big Data (CNN-BD) model designed in this study has the highest fitting degree of 93% and a lowest fitting degree of 78% in the UT-Zap50K dataset. In the Ai2 dataset, the highest fitting degree of the big data classification model is 94%, and the lowest is 76%. In the Kaggle Women’s Shoe dataset, the highest fitting degree of the big data classification model is 92%, and the lowest is 77%. In the Kaggle Men’s Shoe dataset, the highest fitting degree of the big data classification model is 94%, and the lowest is 79%. The designed model has the highest accuracy rate of 93% in sneaker classification, while other models have the highest accuracy rate of around 82% in sneaker classification. Compared with traditional big data technology, the designed model has greatly improved and can adapt to more working environments. This study not only provides technical support for the application of big data technology but also contributes to improving the dissemination effect and promoting the comprehensive development of sneaker consumer culture.

## Introduction

In today’s information society, the continuous development of digitization and big data technology is profoundly changing people’s lifestyles and the direction of socio-economic development. More and more industries are joining the trend of digitization and dataization to provide services and products in a more efficient, accurate, and reliable way. In this digital era, sneakers, as a representative trend culture, lead the development of fashion and youth culture and are gradually accepted and loved by young people [1]. However, with the continuous expansion of the sneaker market and the continuous growth of the consumer group, sneaker consumer culture is no longer just a consumption behavior. Still, it is becoming an important cultural phenomenon with profound social and cultural implications. In sneaker consumer culture, symbolic culture plays an important role. Through disseminating symbolic culture, sneaker consumer culture can not only realize the transmission of commercial value but also influence people’s cultural aesthetics and lifestyles and stimulate their creativity and innovation ability. Therefore, studying how to use digital technology and big data technology to analyze the path of symbolic culture dissemination in sneaker consumer culture, and proposing feasible improvement strategies, is of great significance for promoting the dissemination and development of sneaker consumer culture [2, 3]. Many studies have provided important technical support to promote the development of sneaker culture.

Mariani and Matarazzo (2021) analyzed the connotation and characteristics of big data technology in their study, revealing the opportunities and challenges faced by the dissemination of red culture in the era of big data. They explored feasible paths for innovative dissemination of red culture, proposing to enhance the influence and penetration of red culture dissemination: building a complete red culture network database and constructing a diverse red culture dissemination platform using new technologies. Their aim was to keep the red culture dynamic and highlight its contemporary value [4]. Zhang and Dong (2021) researched the sneaker market through big data analysis, examining the psychological factors, consumer preferences, and evolutionary trends that influence sneaker purchases. They analyzed sneaker comments and rumors on the internet to understand better the characteristics of sneaker consumer culture and behavior [5]. Yu et al. (2021) used a literature review to summarize cultural sharing concepts systematically, the current status of martial arts dissemination, and the application of information technology. They interviewed experts to clarify the feasibility of combining martial arts dissemination with big data and cloud computing under the concept of cultural sharing. Finally, Yu et al. analyzed and summarized data mining and data recommendation technologies of big data and cloud computing, as well as the information sharing platform of the Internet of Things, proposing a strategic vision for martial arts dissemination in the era of "Internet+." They aimed to build a user martial arts behavior database, construct a martial arts information recommendation system based on relevant concepts of recommendation systems, and integrate dissemination channels [6]. Trace and Zhang (2020) used big data analysis of social networks such as Weibo and forums to explore sneaker trends and consumer attitudes toward sneaker brands. In addition to evaluating sneaker appearance and quality, consumers paid great attention to brand symbols, which influenced their brand loyalty and purchase intentions [7]. Yu (2022) addressed the problem that traditional network intrusion detection methods were difficult to implement effectively in intrusion detection due to the complexity, heterogeneity, and large-scale nature of big data. He proposed a hybrid deep learning model based on Convolutional Neural Network and Weight-Dropped Long Short-Term Memory (CNN-WDLSTM) for network intrusion detection in the big data environment. Moreover, he successfully integrated big data and deep learning technologies [8].

The integration of BD and DL technology has reached a high level of maturity, and the discussion on BD technology in cultural communication is extensive. However, the actual application of BD technology in cultural communication is not well-researched. In order to address this gap, this study focuses on the integration of BD technology and sneaker consumption culture to enhance the application and development of BD in cultural communication. Additionally, this study discusses the application of BD technology in the spread of sneaker consumption culture and designs a classification model of cultural products using BD technology and the CNN model. This study provides technical support for improving the effectiveness of sneaker culture communication and contributes to advancing BD technology.

## Sneaker consumption culture based on BD analysis

### Sneaker consumption culture by BD

The fourth industrial revolution, propelled by IoT, BD, robots, and Artificial Intelligence (AI) technologies, is rapidly gaining momentum worldwide. It has propelled humanity into a digital information society based on data [9]. The impact of BD on human life is significant, and its foreseeable development trend is toward serving people. With society’s progressive development strategy, the current and potential applications of BD in various fields should not be underestimated [10].

**Fig 1:**
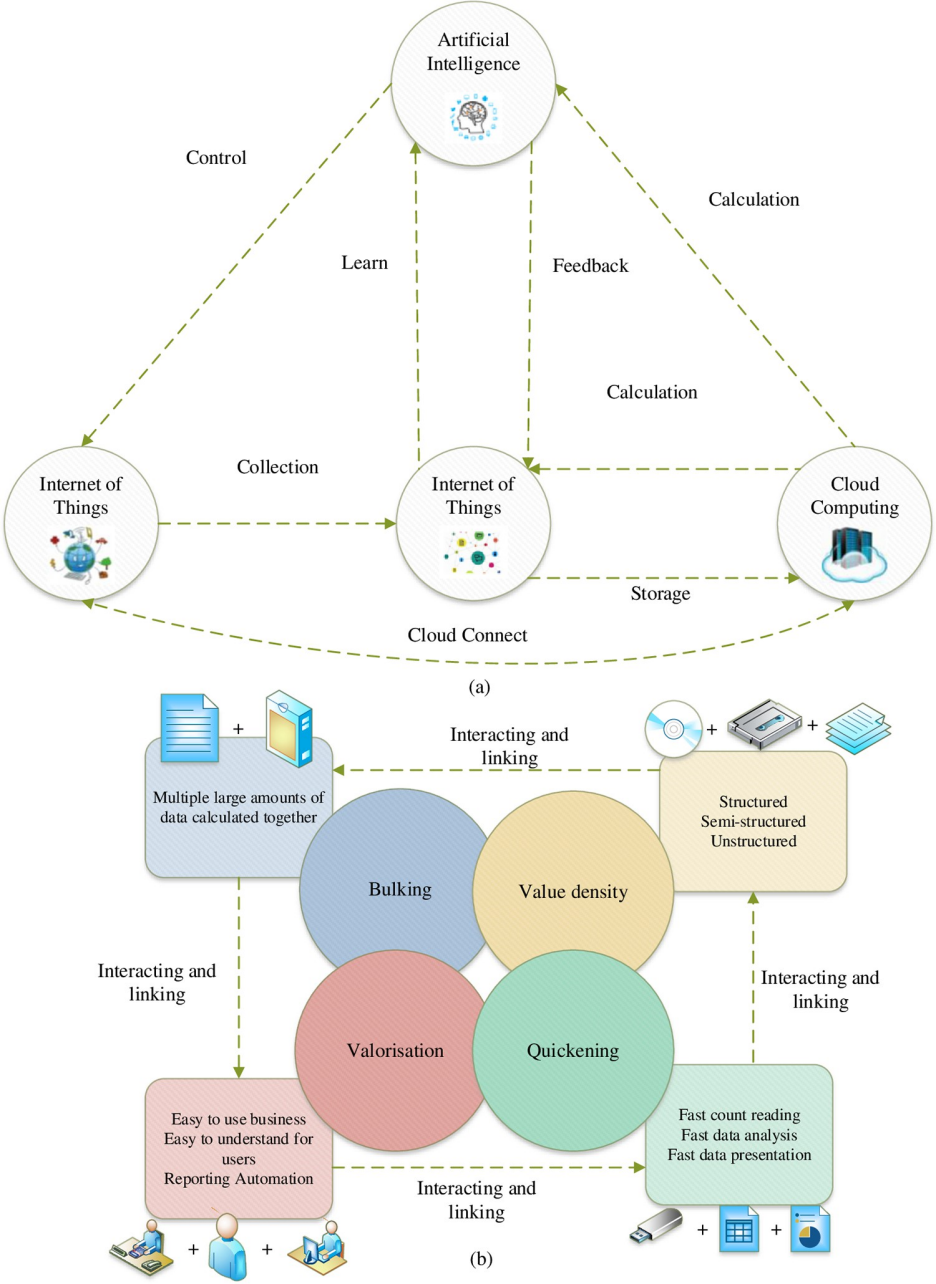
Technical composition and specific characteristics of BD (a is the technical composition; b is the characteristics).

BD, also known as "Big Data," refers to the ability to obtain valuable information from various sources of data. The algorithms and machines used for BD can be applied in cultural communication to track and record audience behavior data of different cultures [11]. Through data mining, it is possible to obtain, store, and analyze audience information, mine useful insights from it, and analyze the needs of different cultures with respect to the audience. This technology can explore effective communication modes for different cultures among the audience under the background of BD. BD is a constantly developing data collection or information asset that is processed using cloud computing, distributed databases, cloud storage, and virtualization technologies. It has penetrated many fields and greatly influenced the development of business forms across various domains. It is characterized by its large quantity, high speed, diversity, and value density [12]. Fig 1 illustrates the development of BD technology and its specific characteristics in cultural dissemination.

Fig 1 depicts four prominent features of BD technology, which are also necessary conditions for data analysis. Firstly, the characteristics of large quantification are exhibited by this technology. The size and variety of data are the most obvious features of BD technology. With the rapid progress of information technology and the explosive growth of data, the era of BD has quietly arrived [13]. Both social networks and mobile networks cannot be separated from the support of data. Various types of data, such as Weibo, text, voice, audio, video, picture, image, and geographical location, have proposed higher standards for the processing capability of this technology [14]. A powerful processing platform and technology are required to present statistics, analysis, and processing through quantification and visualization for the massive data. Secondly, the high-speed feature means that massive data can be created or moved quickly. The internet has the characteristics of timeliness, real-time, and cross-time, which provide a good transmission medium for data [15]. BD relies on the internet as the transmission hub to achieve high-speed efficiency. The rapid development of the internet enables data to be generated, processed, reproduced, and reprocessed at any time and anywhere. BD adapts to the high-speed operation of network data and can instantly obtain valuable information resources from many data so that data can be processed in real-time, which is very popular [16]. Then, the diversity of BD is found in a wide range of information sources. This technology can generate data in various situations and forms. Therefore, multi-channel data sources accelerate the increasing number of personalized data. There are two main categories of data sources. The first category is the automatic recommendation system, which is structured. The system can automatically analyze the user’s behavior trace, access duration, and preference selection and push similar content to the user. The second category is the non-automatic recommendation system, which is not obvious in structure, such as pictures, music, animation, video, etc. These data do not have the capability of automatic recognition and cannot be processed independently. They need to be defined with the help of artificial or AI technology [17]. Finally, the low-value density of BD technology is a potential deficiency of this technology. Data value is controlled from the macro level, which inevitably has a low-value density but can still understand the data value as a whole [18]. There are inevitably many good and bad network information. Users’ valuable data cannot be separated from BD’s value judgment and classification [19]. BD can analyze and predict users’ needs, filter out irrelevant data, and mine valuable data. Machine learning, AI, data mining, and other methods are used to obtain valuable data that users need, play BD’s role in various application fields, and highlight its functional advantages [20].

### Symbolic culture communication of sneaker consumption culture

The emergence of sneaker culture is closely related to the growth and development of basketball culture. Basketball, born in 1891, has been imbued with the significance of competition, faith, interests, stories, good and evil, love and hate, and emotions since its inception. The growing number of young basketball enthusiasts has provided a mass base for the formation of shoe culture [21]. In basketball, sneakers are the most crucial sports equipment. Fans keenly observe the shoes worn by players during each game. Each sneaker style has its own legend and is highly popular with fans [22]. In recent times, people’s interest in sneakers has shifted from practicality to aesthetics. With the improvement in people’s economic status, fans’ pursuit of aesthetic appreciation of sneakers has become diverse. The commercial aspects of sneaker culture have become increasingly vital, and its elements have gradually become indispensable [23].

Due to their association with basketball culture, sneakers have gained popularity among many people. As a result, a growing number of young people have become involved in the practice, forming the core of sneaker culture [24]. Different groups have varying needs for sneakers, such as appearance, functionality, and durability [25]. However, the passionate consumption of young people has given rise to some irrational factors in the sneaker culture, where sneakers have come to symbolize their own identities. Furthermore, the emergence of blind consumption is a new trend created by advertising and the media [26]. Many individuals have criticized the consumerism associated with the sneaker culture, and the rapid expansion of this culture has encouraged symbolic consumption from the outset. This criticism has brought the issue of cultural development and business to the forefront and sparked a debate between youth culture and business [27].

The dissemination of sneaker culture largely relies on the transmission of symbolic culture. Cultural semiotics, which interpret culture as symbols, is not only an academic approach to understanding culture but also assists in defining its essential characteristics [28]. However, the main challenge faced by the extensive sneaker culture system is the appropriate design of the symbolic culture transmission process. Therefore, the optimization of the transmission of sneaker symbol culture through science and technology is a plausible technical solution.

### Design of cultural communication model of intelligent symbols under BD technology

BD and DL technologies can be used to optimize the transmission of symbolic culture in sneaker consumption culture for better transmission effects. One of the most effective methods to achieve this is to classify the symbolic culture, and CNN can be used to optimize this process. CNN can aid in symbol classification through feature extraction and final output. Therefore, this paper utilizes CNN to optimize the classification of symbolic culture [29]. The process of data feature extraction can be expressed using Eq (1):

$$F_{i}=f(G_{i⊗}F_{i-1}+b_{i})$$ (1)

In Eq (1), ***i*** represents the network convolution level. **G** represents the calculated weight. **b** represents the offset vector in the calculation process. The excitation function is used to obtain the eigenvector **F**_***i***_. The calculation of CNN’s pooling process is shown in Eq (2):

$$F_{i}=subsampling(F_{i-1})$$ (2)

After multiple rounds of pooling and a fully connected network, the extracted features are then represented and classified. The mapping results can be expressed as shown in Eq (3):

$$Y(m)=P(L=l_{m}|F_{0};(G,b))$$ (3)

In Eq (3), ***m*** represents the index of the label category. **L** is the loss function. ***P*** represents a mapping operation. The loss function is calculated as shown in Eqs (4) and (5):

$$N(G,b)=-\sum_{m=1}^{\left|Y\right|}log\;Y(m)$$ (4)

M(G,b)=1|Y|∑m=1|Y|(Y(m)−Y^(m))2 (5)

In order to reduce the overfitting of network parameters, a two-norm term is usually added to the final loss function. The calculation of the two-norm term is shown in Eqs (6)–(8):

$$E(G,b)=L(G,b)+\frac{\lambda }{2}G^{T}G$$ (6)

$$G_{i}=G_{i}-\eta \frac{\partial E(g,b)}{\partial g_{i}}$$ (7)

$$b_{i}=b_{i}-\eta \frac{\partial E(g,b)}{\partial b_{i}}$$ (8)

**Fig 2:**
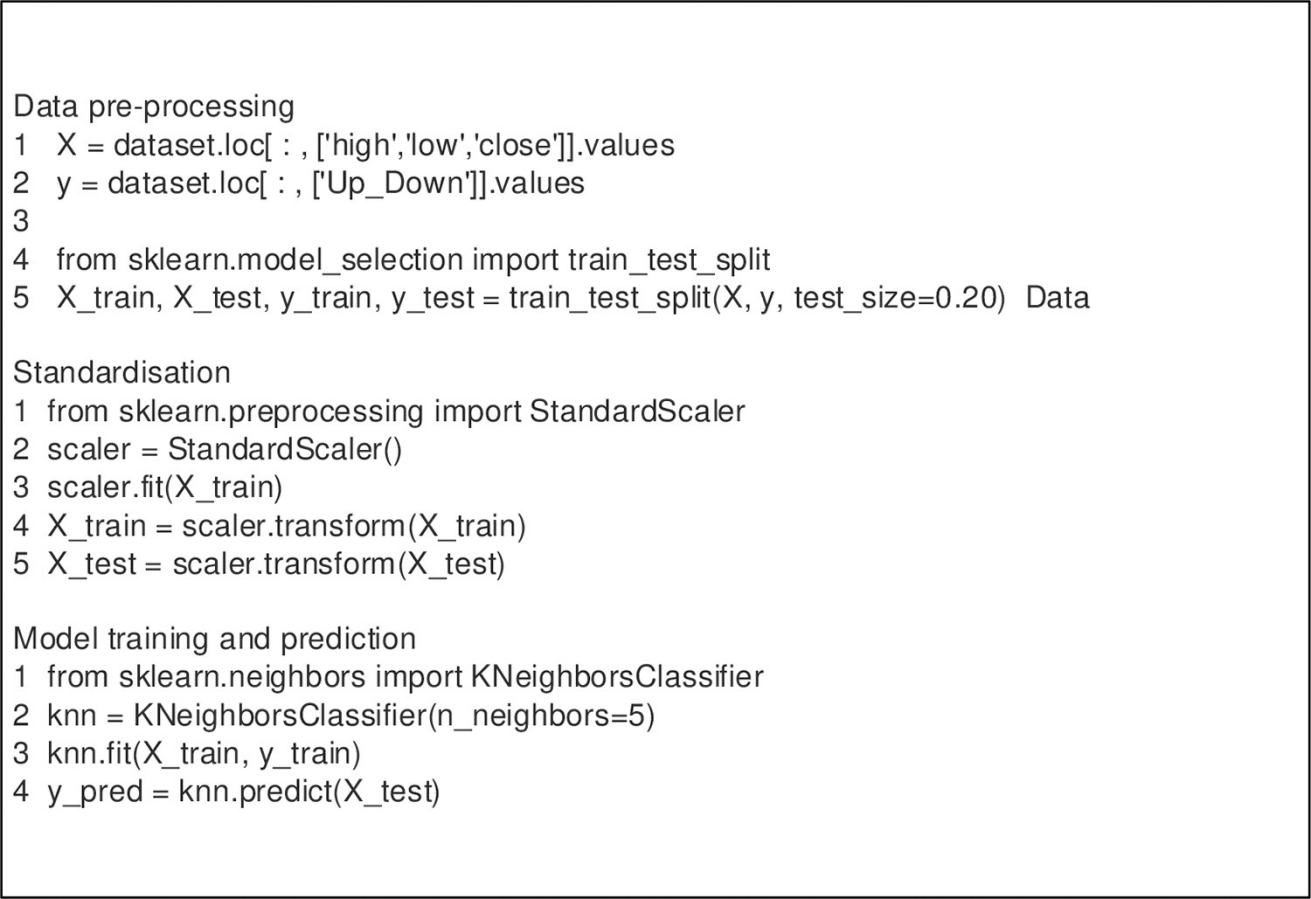
Classification algorithm of BD.

Eqs (6)–(8) depict the classification process of symbol culture implemented by CNN technology, which provides significant support for later integration of audience groups and symbol culture using BD technology and attains a high degree of intelligence. Here, ***η*** represents the learning rate [30]. This paper utilizes BD technology to accomplish this objective. Fig 2 illustrates the data preprocessing and standardization algorithm of BD utilized for symbol culture classification.

In Fig 2, the optimization of the classification algorithm and the classification effect of symbol culture is designed to improve the communication effect of sports shoe consumption symbol culture under BD technology, and CNN technology is used to provide users with better choices [31]. The CNN-BD model is developed to achieve an innovative integration of traditional CNN and BD technology models, providing important technical support for disseminating sneaker symbol culture.

### Design of communication data of symbolic culture

**Fig 3:**
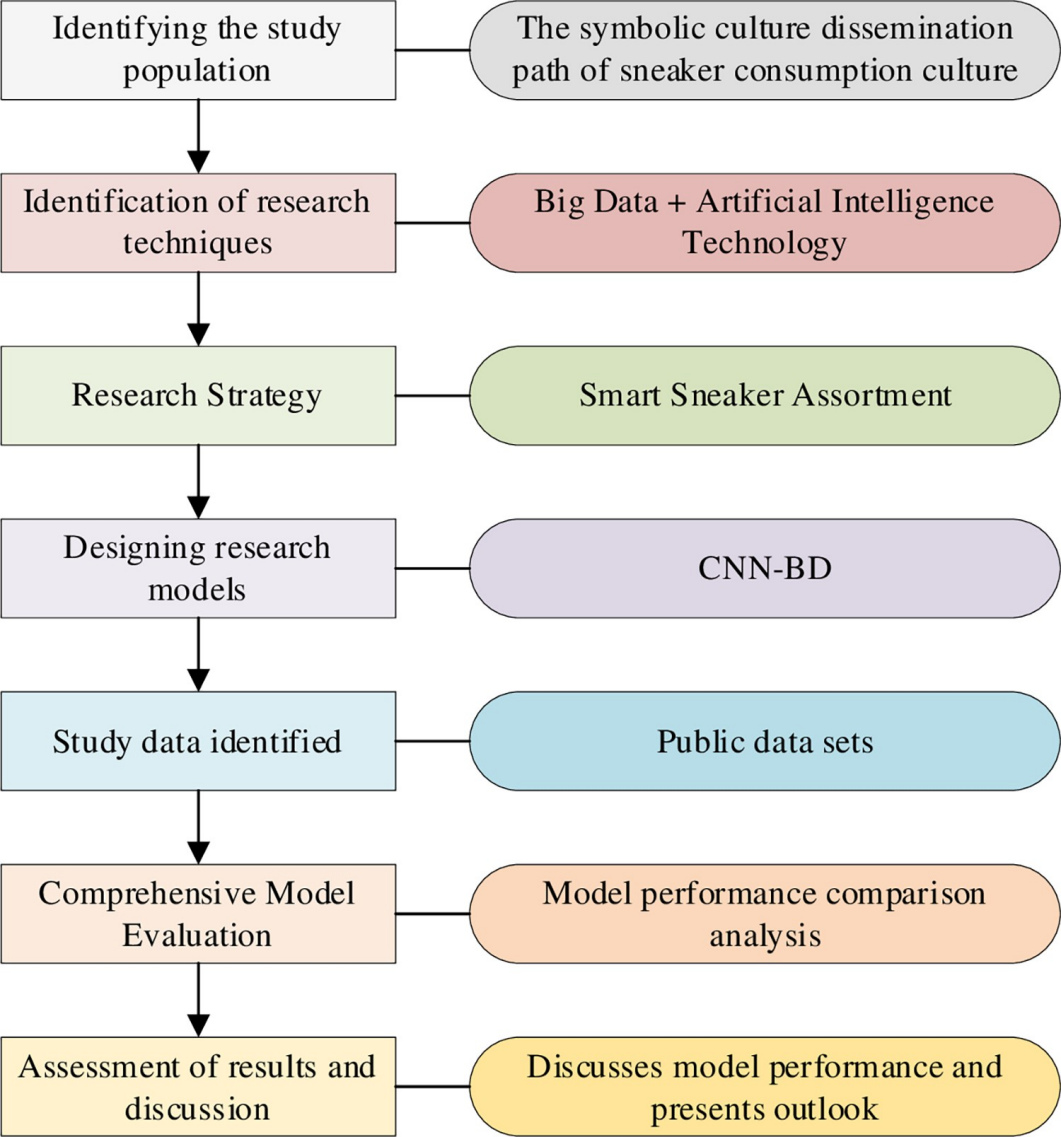
Research process.

This paper aims to evaluate the design model by utilizing a dataset to classify sneakers based on their color, size, purpose, brand, shape, and material in order to explore the model’s classification effect on different types of shoes and provide technical guidance for the sneaker culture process. The dataset comprises four sources: the UT-Zap50K dataset includes 50025 catalog images of shoes, classified into shoes, sandals, slippers, and boots. The images are captured on a white background, with the shoes centered and photographed in the same direction to facilitate analysis. The Ai2 dataset labels and classifies shoes using Diagram Part Labeling (DiPART). DiPART is a chart dataset that provides point supervision part notes. The Kaggle women’s shoe dataset contains 10,000 images and information about women’s shoes, while the Kaggle men’s shoe dataset is from the same source and includes 10,000 images of men’s shoes and their information. The model is analyzed using these four datasets to explore its classification effect. Fig 3 shows the research flow.

Table 1 shows the experimental environment.

**Table 1:** Design of research environment.

Number	Equipment	Parameter
1	Processor	Intel(R) Core (TM) i5-7300HQ CPU @ 2.50GHz
2	Memory	8.00 GB
3	Operating system	Windows 10×64
4	Operating platform	MATLAB 9.2.0.538062 (R2017a)

## Communication analysis of sneaker consumption symbol culture by the BD analysis

### Classification effect evaluation of sneaker symbol culture by BD technology

**Fig 4:**
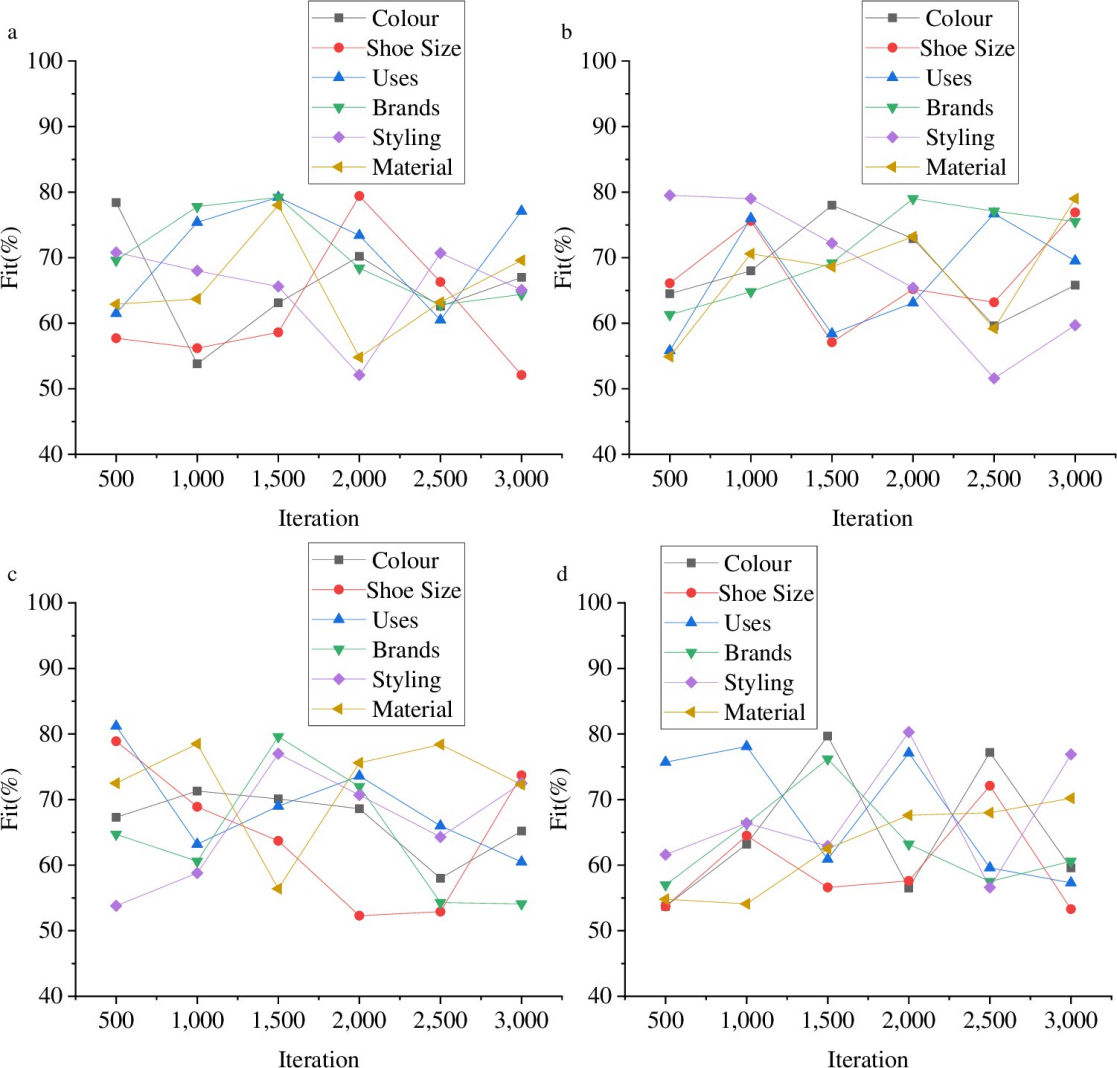
Evaluation of the CNN-BD data analysis performance (a is UT-Zap50K data set; b is Ai2 data set; c is Kaggle woman’s shoe data set; d is Kaggle man’s shoe data set).

In order to enhance the development of sneaker consumption culture and improve the communication effect of its symbolic culture, this paper optimized the BD classification algorithm by utilizing CNN technology. Subsequently, a model was designed to classify the symbolic culture of sneaker consumption, providing technical support for enhancing the consumption level of sneakers. In order to evaluate the classification performance of the model, various data sets were utilized. Fig 4 depicts the evaluation of the data analysis performance of sneaker consumption symbol culture via BD technology.

**Fig 5:**
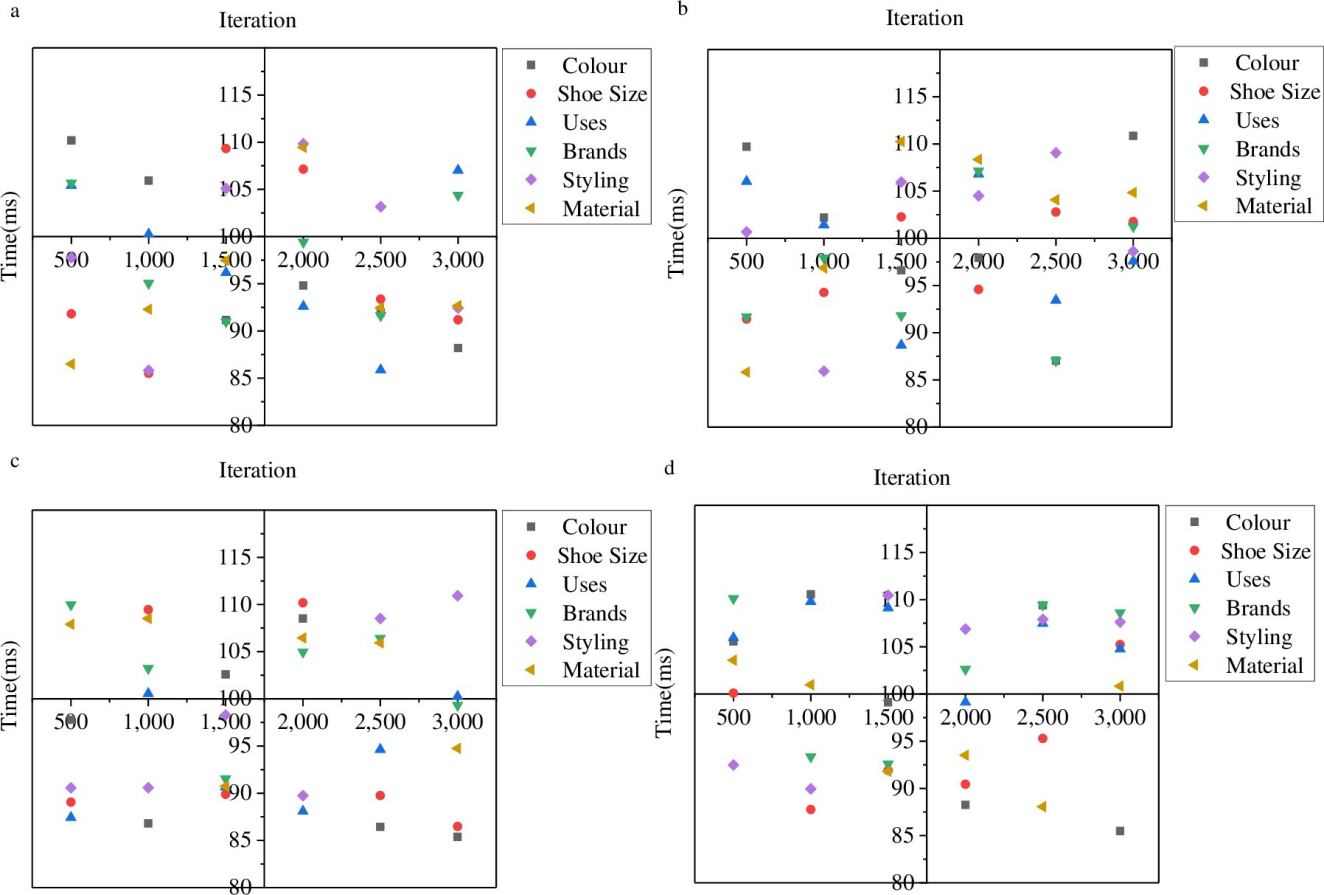
Evaluation of model data labeling accuracy (a is UT-Zap50K dataset, b is Ai2 dataset, c is Kaggle woman’s shoe dataset, d is Kaggle man’s shoe dataset).

In Fig 4, the performance of the BD classification algorithm is evaluated, indicating that the accuracy of the BD classification algorithm is low. The UT-Zap50K dataset shows that the model’s highest fitting degree is 81%, while the lowest is 57%. In the Ai2 dataset, the fitting degree of the BD classification model ranges from 80% at the highest to 52% at the lowest. For the Kaggle women’s shoe dataset, the highest fitting degree of the model is 83%, and the lowest is 54%. The Kaggle men’s shoe dataset indicates that the model’s highest fitting degree is 81%, and the lowest is 54%. Fig 5 provides an analysis of the model’s classification efficiency.

In Fig 5, the model’s working efficiency is tested and evaluated. The research findings show that the maximum and minimum data processing times for the UT-Zap50K data set are 112ms and 86ms, respectively. For the Ai2 data set, the maximum and minimum data processing times are 110ms and 87ms, respectively. The maximum and minimum data processing times for the Kaggle women’s shoe data set are 112ms and 84ms, respectively. Finally, the longest and shortest data processing times for the Kaggle men’s shoe data set are 111ms and 86ms, respectively. Consequently, the designed model exhibits excellent data processing efficiency, although its fitting degree is not high. Therefore, this paper utilizes CNN technology to optimize the model’s performance.

### Performance evaluation of BD optimization model

**Fig 6:**
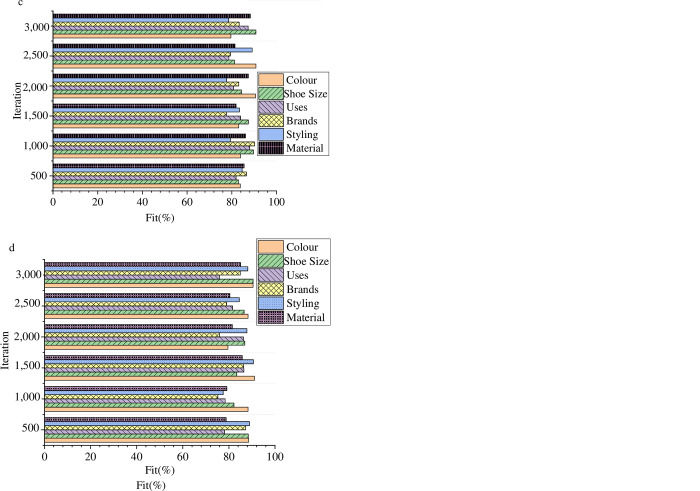
Comparison results of model performance (a is UT-Zap50K dataset; b is Ai2 dataset; c is Kaggle woman’s shoe dataset; d is Kaggle man’s shoe dataset).

The model’s classification performance on sneaker consumption symbol culture is evaluated in four datasets to investigate its practical working efficiency. Following the experimental design, the classification effect of the model is analyzed based on six aspects and compared with the results to explore the comprehensive performance of the designed model. Fig 6 presents the evaluation result of the classification effect of the symbolic cultural products of model sneakers.

**Fig 7:**
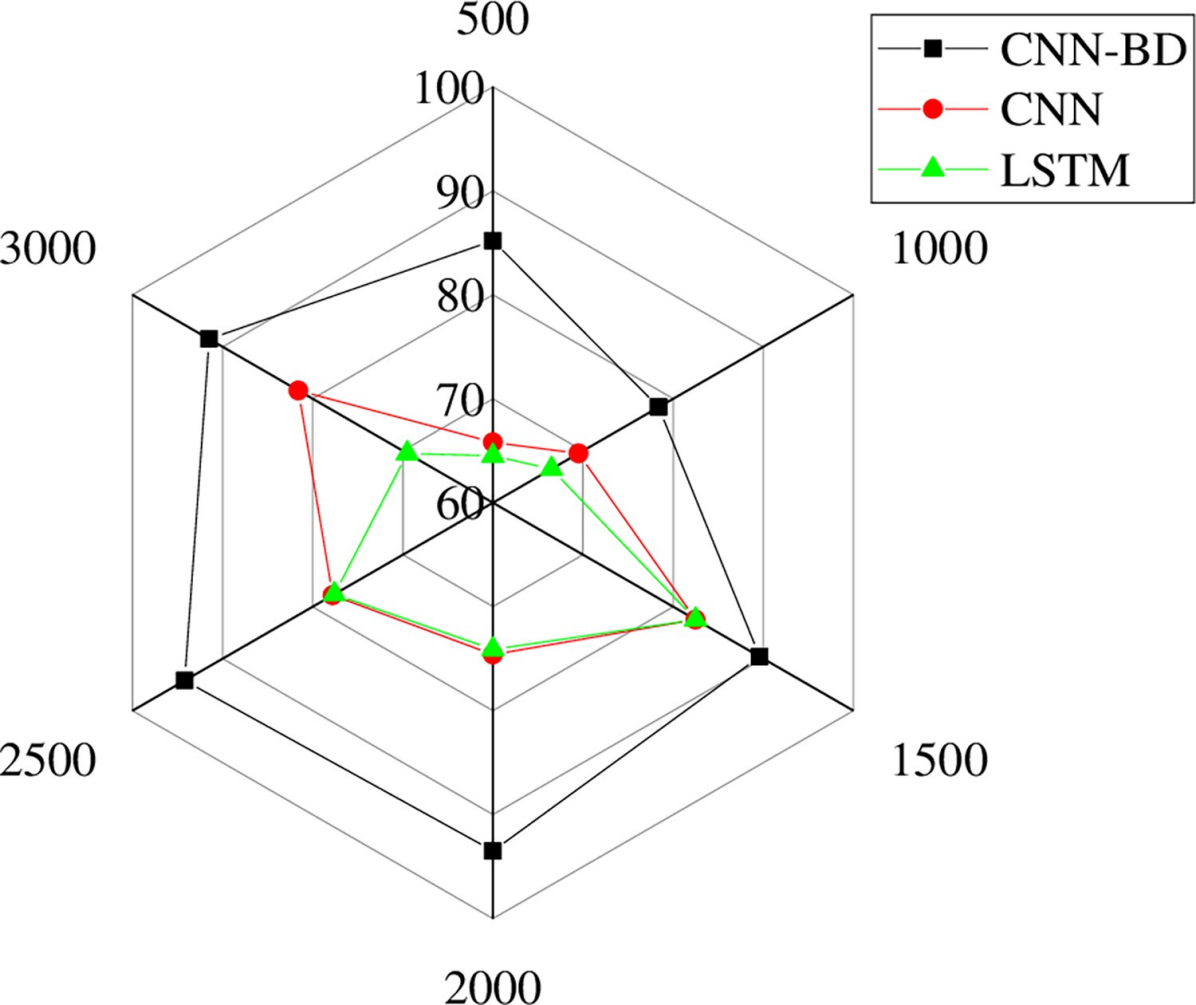
Comparison of model performance.

In Fig 6, this paper optimizes the big data model using a Convolutional Neural Network (CNN) and evaluates its comprehensive performance. The results demonstrate that the CNN-BD model designed in this paper has the highest fitting degree of around 93% in the UT-Zap50K dataset and the lowest at around 78%. In the Ai2 dataset, the highest fitting degree of the big data classification model is around 94%, and the lowest is around 76%. In the Kaggle woman’s shoe dataset, the highest fitting degree of the big data classification model is around 92%, and the lowest is around 77%. In the Kaggle man’s shoe dataset, the highest fitting degree of the big data classification model is around 94%, and the lowest is around 79%. The optimized model designed in this paper has greatly improved in comparison to traditional big data techniques. In Fig 7, to highlight the research model’s advantages, the designed model is compared with CNN and Long Short-Term Memory (LSTM) models to explore the comprehensive performance of the research model.

The model designed in Fig 7 has the highest accuracy in sneaker classification, ranging from around 93% to 78%, while other models have an accuracy range of around 82% to 64%. The model designed in this paper has greatly improved the accuracy of sneaker classification.

## Conclusion

Science and technology exert a profound impact on the advancement of society, particularly BD technology. In order to expand the influence of BD technology and enhance its effectiveness in cultural communication, BD technology is used to optimize the transmission path and strategy of the symbolic culture of sneaker consumption culture. In contrast, DL technology is utilized to optimize the BD technology model and improve the communication effect of sneaker consumption symbol culture. The paper introduces the application concept of BD technology in cultural communication. It discusses the fundamental concept of symbol culture and communication based on BD technology support for the consumption culture of sneakers. Subsequently, the paper designs and evaluates the classification model of sneaker consumption symbol cultural products based on CNN and BD technology. The results show that the designed model’s maximum data processing time is 112ms in UT-Zap50K, and the minimum is 86ms. In Ai2, the maximum data processing time is 110ms, and the minimum is 87ms. In Kaggle woman’s shoes, the maximum data processing time is 112ms, and the minimum is 84ms. In Kaggle man’s shoe, the longest data processing time is 111ms, and the shortest is 86ms. The CNN-BD model designed has the highest fitting degree of 93% in the UT-Zap50K dataset. In the Ai2 dataset, the fitting degree of the BD classification model is up to 94%. In the Kaggle woman’s shoe dataset, the fitting degree of the BD classification model is up to 92%. In the Kaggle man’s shoe dataset, the fitting degree of the BD classification model is up to 94%. The designed optimization model has been significantly improved in terms of data technology. Although this paper provides a more comprehensive design concept of the technical model and evaluates the model, the specific research on the development strategy of sneaker culture is not flawless. Hence, in future research, this project will enhance the research of progress strategies, promote the development of sneaker culture, and improve the effect of cultural communication.

## Supporting information

S1 Data (ZIP)

S1 Text (DOCX)
