# Supplementary material for: The communication path and improvement strategy of symbolic culture of sneaker consumption culture using the big data analysis
Source: PLoS One. 2023 Jul 19;18(7):e0287757. doi: 10.1371/journal.pone.0287757 (PMC10355455; doi:10.1371/journal.pone.0287757)
Supplement: S1 Text — (DOCX) [file pone.0287757.s002.docx]

The following is a code for an image classification model that utilizes convolutional neural networks to optimize big data technology:

import tensorflow.keras as keras

# convolutional neural network is used to construct deep learning model

model = keras.Sequential([

keras.layers.Conv2D(32, (3,3), activation='relu', input_shape=(28, 28, 1)),

keras.layers.MaxPooling2D((2,2)),

keras.layers.Conv2D(64, (3,3), activation='relu'),

keras.layers.MaxPooling2D((2,2)),

keras.layers.Conv2D(64, (3,3), activation='relu'),

keras.layers.Flatten(),

keras.layers.Dense(64, activation='relu'),

keras.layers.Dense(10, activation='softmax')

])

# Loading training data and test data,and preprocess the data

train_images, train_labels = load_train_data()

test_images, test_labels = load_test_data()

train_images = train_images / 255.0

test_images = test_images / 255.0

# training model

model.compile(optimizer='adam', loss='sparse_categorical_crossentropy', metrics=['accuracy'])

model.fit(train_images, train_labels, epochs=5, validation_data=(test_images, test_labels))

# Evaluation model performance

test_loss, test_acc = model.evaluate(test_images, test_labels)

print('Test accuracy:', test_acc)

This model uses convolutional neural networks to classify images, including 32 convolutional kernels with a size of (3,3) and 64 convolutional kernels with a size of (3,3), as well as a max pooling layer and fully connected layer. The model requires loading training and test data and preprocessing the data, such as normalizing pixel values by dividing them by 255. Finally, the model is trained and evaluated on the test set through compilation, fitting, and evaluation to obtain accuracy.

The following is an example code for a shoe symbol classification model based on CNN.

# Import the required libraries

import numpy as np

import tensorflow as tf

# Read Dataset

def load_dataset():

# Load training and testing data

train_data = np.load("train_data.npy")

train_labels = np.load("train_labels.npy")

test_data = np.load("test_data.npy")

test_labels = np.load("test_labels.npy")

# Normalized data

train_data = train_data.astype(np.float32) / 255.

test_data = test_data.astype(np.float32) / 255.

return train_data, train_labels, test_data, test_labels

# modeling

def build_model():

# Define Input

inputs = tf.keras.layers.Input(shape=(28, 28, 1))

# Convolutional Layer

x = tf.keras.layers.Conv2D(32, (3,3), activation='relu', padding='same')(inputs)

x = tf.keras.layers.MaxPooling2D(pool_size=(2, 2))(x)

# Convolutional Layer

x = tf.keras.layers.Conv2D(64, (3,3), activation='relu', padding='same')(x)

x = tf.keras.layers.MaxPooling2D(pool_size=(2, 2))(x)

# Convolutional Layer

x = tf.keras.layers.Conv2D(128, (3,3), activation='relu', padding='same')(x)

x = tf.keras.layers.MaxPooling2D(pool_size=(2, 2))(x)

# Dropout storey

x = tf.keras.layers.Dropout(0.5)(x)

# Fully connected layer

x = tf.keras.layers.Flatten()(x)

x = tf.keras.layers.Dense(512, activation='relu')(x)

# Dropout storey

x = tf.keras.layers.Dropout(0.5)(x)

# Output layer

outputs = tf.keras.layers.Dense(10, activation='softmax')(x)

# efinition model

model = tf.keras.Model(inputs, outputs)

return model

# training model

def train_model():

# Load Dataset

train_data, train_labels, test_data, test_labels = load_dataset()

# modeling

model = build_model()

# Compilation Model

model.compile(optimizer='adam', loss='sparse_categorical_crossentropy', metrics=['accuracy'])

# training model

model.fit(train_data, train_labels, batch_size=32, epochs=10, validation_data=(test_data, test_labels))

# Save Model Weights

model.save_weights("shoe_symbol_classification_model.h5")

if __name__ == "__main__":

train_model()

The provided code employs a deep convolutional neural network with three convolutional layers, three pooling layers, and two fully connected layers to train the model for shoe symbol classification. Before training, it is necessary to load the shoe symbol classification dataset and normalize it. Then, using the Keras API in the TensorFlow framework, the model is built and compiled, using cross-entropy as the loss function and Adam as the optimizer. The model is fitted with training data. Finally, the trained model weights are saved.

The last part is a shoe classification model optimized based on convolutional neural networks and big data technology, which is used to train a convolutional neural network-based shoe symbol classifier. The dataset used is UT-Zap50K. This code is implemented using PyTorch, which includes downloading and preprocessing the dataset.

Firstly, it is necessary to install the PyTorch and torch-vision libraries.

pip install torch torchvision

Then, the experiment downloads and preprocess the UT-Zap50K dataset:

import torch

import torchvision

import torchvision.transforms as transforms

# Define preprocessing methods

transform = transforms.Compose(

[transforms.Resize((224, 224)), # Resize the image to 224x224

transforms.ToTensor(), # Convert to Tensor data type

transforms.Normalize(mean=[0.485, 0.456, 0.406], std=[0.229, 0.224, 0.225])]) # Standardize the image to ensure that the mean of each pixel is 0 and the standard deviation is 1

# Download and load dataset

train_dataset = torchvision.datasets.ImageFolder(root='ut-zap50k-images',

transform=transform)

train_loader = torch.utils.data.DataLoader(train_dataset, batch_size=64,

shuffle=True, num_workers=2)

Next, a convolutional neural network model is created, and cross-entropy is used as the loss function.

import torch.nn as nn

import torch.nn.functional as F

class Net(nn.Module):

def __init__(self):

super(Net, self).__init__()

self.conv1 = nn.Conv2d(3, 16, 3, padding=1)

self.conv2 = nn.Conv2d(16, 32, 3, padding=1)

self.conv3 = nn.Conv2d(32, 64, 3, padding=1)

self.pool = nn.MaxPool2d(2, 2)

self.fc1 = nn.Linear(64 * 28 * 28, 512)

self.fc2 = nn.Linear(512, 10)

def forward(self, x):

x = self.pool(F.relu(self.conv1(x)))

x = self.pool(F.relu(self.conv2(x)))

x = self.pool(F.relu(self.conv3(x)))

x = x.view(-1, 64 * 28 * 28)

x = F.relu(self.fc1(x))

x = F.dropout(x, training=self.training)

x = self.fc2(x)

return x

net = Net().cuda()

criterion = nn.CrossEntropyLoss()

Finally, the model is trained using the stochastic gradient descent algorithm, and the model is saved after each epoch.

import torch.optim as optim

optimizer = optim.SGD(net.parameters(), lr=0.001, momentum=0.9)

for epoch in range(10): # Train 10 epoch

running_loss = 0.0

for i, data in enumerate(train_loader, 0):

inputs, labels = data

inputs, labels = inputs.cuda(), labels.cuda()

optimizer.zero_grad()

outputs = net(inputs)

loss = criterion(outputs, labels)

loss.backward()

optimizer.step()

running_loss += loss.item()

if i % 200 == 199:

print('Epoch %d, Batch %d Loss: %.3f' %

(epoch+1, i+1, running_loss/200))

running_loss = 0.0

# Save a model

PATH = 'shoe_classifier.pth'

torch.save(net.state_dict(), PATH)

The model uses a simple convolutional neural network with three convolutional layers and two fully connected layers. Training the model on the UT-Zap50K dataset takes several hours, so only ten epochs are used for demonstration. If a higher classification accuracy is desired, the number of epochs can be increased, the model architecture can be optimized, or the size of the training dataset can be increased.
